# Effect of the drinker model on growth performance, water use and manure volume for piglets during the nursery phase

**DOI:** 10.1093/tas/txaf084

**Published:** 2025-07-18

**Authors:** Gustavo Schlindwein, Sarah Ribeiro Krasilchik, Natalia Rigo, Caroline Pellis, Juliana Bona Preisler, Natália Rampon Cendron, José Cristani, Sandra Davi Traverso

**Affiliations:** Laboratory of Animal Health and Production, Santa Catarina State University (UDESC) 88520-000 Lages, Brazil; Laboratory of Animal Health and Production, Santa Catarina State University (UDESC) 88520-000 Lages, Brazil; Laboratory of Animal Health and Production, Santa Catarina State University (UDESC) 88520-000 Lages, Brazil; Laboratory of Animal Health and Production, Santa Catarina State University (UDESC) 88520-000 Lages, Brazil; Laboratory of Animal Health and Production, Santa Catarina State University (UDESC) 88520-000 Lages, Brazil; Laboratory of Animal Health and Production, Santa Catarina State University (UDESC) 88520-000 Lages, Brazil; Laboratory of Animal Health and Production, Santa Catarina State University (UDESC) 88520-000 Lages, Brazil; Laboratory of Animal Health and Production, Santa Catarina State University (UDESC) 88520-000 Lages, Brazil

**Keywords:** hydration, nursery piglets, sustainability

## Abstract

The water fasting period and the volume of water wasted during the nursery phase are directly related to the type of water fountain used. Therefore, this study aimed to compare different water fountain models regarding total water consumption, waste production, and growth performance of piglets on a commercial farm during the nursery phase. A total of 1,104 animals were divided into 3 experimental treatments: Treatment 1—Automatic bowl drinker (ABD2) model containing 2 drinkers per pen. Treatment 2 - (ABD4) the same model as T1, but with 4 drinkers per pen. Treatment 3—Swing nipple drinker (SND4) model with 4 drinkers per pen. Water usage at the drinkers (L/pig/day) was higher in SND4 than in ABD2 and ABD4 (*P* < 0.0001). The water-to-feed ratio was higher in SND4 than in ABD2 and ABD4 (*P* < 0.0001). The water-to-weight gain ratio was also higher in SND4 than in ABD2 and ABD4 (*P* < 0.0001). Regarding waste production, ABD2 and ABD4 presented lower volumes than SND4 (*P* < 0.0001). Additionally, liquid manure in ABD2 and ABD4 had a higher total solids content than SND4 (*P* < 0.0001). The coefficient of variation in body weight at the end of the experiment was lower in ABD4 than in SND4 (*P* < 0.05) and equal to ABD2 (*P* > 0.05). Daily weight gain was higher in SND4 than in ABD2 (*P* < 0.05) and equal to ABD4 (*P* > 0.05). The ABD used in this study resulted in lower total water consumption, and consequently lower manure volume produced. Additionally, the animals reached their final weight more uniformly in the pen, and there was no difference in growth performance when the number of animals per drinker was the same among treatments.

## Teaser

Switching to bowl drinkers cut water waste by 74% without sacrificing growth, a win for both pigs and the planet. This study proves sustainable farming starts with smarter hydration—less waste, better performance, and more uniform piglets.

## INTRODUCTION

Water is a major component of the swine body, as it is for other living beings (Shields et al., 1983). However, water and its consumption are often neglected in the swine production chain (Thulin and Brumm, 1991). Piglets can take up to 2 d after weaning to learn how to drink water and eat normally (Brooks et al., 1984), and water deprivation during the first 24 h post-weaning negatively impacts their performance both in the short and long term. Dehydration can be a primary cause of stunted growth and poor performance within a batch (Horn et al., 2014). Proper hydration primarily depends on factors such as water quality, environment (Brumm, 2006), animal health (McGlone and Pond, 2003), and the type of drinker used (Torrey et al., 2008).

The drinker model can affect water consumption (Magowan et al., 2007) and water wastage, which, in turn, increases the volume of waste produced (Brumm et al., 2000; Andersen et al., 2014). Consequently, this leads to higher costs for the storage, transport, distribution, and treatment of wastewater (Oliveira, 2002). Optimizing water consumption in piglets during the first hours post-weaning, reducing total water usage, and consequently decreasing waste production are essential for a more sustainable production chain.

In nursery facilities, the main types of drinkers used are swinging nipple drinkers, fixed nipple drinkers, and bowl drinkers, ranging from nipple models with a reservoir to float-level bowl models. This study compared an automatic constant-level bowl drinker with a swinging nipple drinker regarding zootechnical performance and waste volume produced by piglets during the nursery phase.

## MATERIALS AND METHODS

The experiment was conducted in a commercial nursery with a housing capacity of 5,000 animals, located in the midwestern region of the state of Santa Catarina, Brazil (27° 9’ 11’“ S, 51° 43” 41’‘ W), between January and February. The maximum and minimum temperatures recorded inside the facility during the experiment were 32.6°C and 19.6°C, respectively.

### Animals and Management

All procedures and handling in this study were approved by the CEUA—Animal Ethics Committee of UDESC (Universidade do Estado de Santa Catarina), process no. 3159191222. A total of 1,104 weaned piglets (male and female) were used (PIC 337 x GA2030; Pig Improvement Company, SC/Brazil, and Genética Aurora, SC/Brazil) with an average age of 28.32 d and an average weight of 8.2 ± 1.15 kg during the 35-day period. The animals had access to feed through creep feeding management starting at 7 d of age until weaning. At weaning, they were vaccinated for PCV2 and *Mycoplasma hyopneumoniae*. No animals needed to be removed during the experimental period.

### Experimental Desgin

The animals were divided into 24 elevated pens with slatted plastic flooring, each measuring 3.70 × 3.58 meters, totaling 46 animals per pen, adhering to 0.28 m²/animal as per Normative Instruction No. 113 (MAPA, 2020). International standards corroborate the adequacy of this density: the European Union requires at least 0.15 m² for pigs ≤ 10 kg, 0.20 m² for 10 to 20 kg, and 0.30 m² for 20 to 30 kg (European Commission, 2008), while the Canadian Code of Practice recommends 0.24 to 0.32 m² for pigs 18 to 30 kg housed on slatted floors (NFACC, 2023). Consequently, the floor area adopted in the present experiment is fully compliant with both national and widely cited international guidelines. The animals were distributed across 3 experimental treatments: Treatment 1- Automatic bowl drinker (ABD2)—model with a water level valve, with 46 animals for 2 drinkers; Treatment 2—the same model as T1, but with 46 animals for 4 drinkers (ABD4); and Treatment 3—Swinging nipple drinker model (SND4), with 46 animals for 4 drinkers. For each treatment, 4 pens of males and 4 pens of females were used. The nipple drinkers used were Chupetas Inox Creche (Corti Avioeste, Cunha Porã, SC), installed 2 per swinging nipple drinker. The height was adjusted daily to 5 cm above the back of the smallest piglet in the pen and the average flow rate was 1.216 ± 0.257 mL/min. The bowl drinkers used were Habitat® CDD Arco Inox and Habitat® CDD Arco Inox Duplo (Magnani, Seara, SC), and were installed along the central line of the pen. These drinkers feature a pressure valve that maintains a constant 2 cm water layer for consumption.

### Diet

3 feeding phases were used, composed as follows: Phase 1: 2.5 kg of feed per animal with 3,450 kcal/kg of metabolizable energy, 21.5% crude protein, and 1.45% digestible lysine. Phase 2: 4 kg of feed per animal with 3,437 kcal/kg of metabolizable energy, 21.6% crude protein, and 1.42% digestible lysine. Phase 3: Used until the 35th d of housing, with 3,420 kcal/kg of metabolizable energy, 21% crude protein, and 1.36% digestible lysine.

### Total Water Consumption, Manure Volume and Total Solids Content

To evaluate total water consumption, 2 water meters were installed per pen: one at the water inlet for the drinker and another at the water inlet for the feeding trough. Water meter readings were taken daily at 7:00 AM and 7:00 PM. Water consumption from 7:00 AM to 7:00 PM was classified as daytime consumption, and from 7:00 PM to 7:00 AM as nighttime consumption. Before housing the animals, the water meters were tested using containers with known volumes and replaced if the measurement error exceeded 5%.

The average volume of the slurry pits in the pens used for the experiment was calculated to determine the amount of manure produced per animal. Each slurry pit stored waste produced from 2 pens. For this reason, treatments were distributed across every 2 pens so that each pit was shared between the same treatments. A mark was made on all pits to indicate a volume of 4,542 liters. The slurry level in the pits was checked daily, and when it reached the marked level, the waste was discharged into the manure storage area, and the volume produced was recorded. On the 35th d, the slurry heights were measured at 5 different points in each pen, and the final volume was calculated and added to the total.

For the quantification of total solids, 4 liters of slurry were collected from each experimental pen and analyzed according to the methodology described by Apha (1999), Standard Methods, 2,450 Solids - 2450G. The following formula was used to calculate total solids (%): Total solids (%) = (P1—P0) × 100 / sample volume (L), where P1 (g): weight of the dry residue in the oven + crucible and P0 (g): weight of the crucible.

### Growth Performance

The animals were ear-tagged and individually weighed. 3 weighings were conducted: on day 0 (housing), at 16 d, and at 35 d of housing. These served for analyses of average daily gain (ADG), feed conversion ratio (FCR), and coefficient of variation (CV) of weight, where each pen was considered an experimental unit. Feed consumption per pen was measured to calculate daily feed intake. For the calculation of water-to-feed and water-to-weight-gain ratios, total water usage was the sum of water consumed through the drinkers and feeding troughs.

The animals were monitored daily, and any acute clinical signs were deemed eligible for injectable treatment, which was administered according to the medication’s package insert recommendations. Mortalities and medical interventions were recorded, and each injectable protocol administered was considered a single unit of medical intervention.

### Statistical Analisis

The data were analyzed using the Jamovi software—version 2.2.5 (The jamovi project, 2021). Normality was tested using the Shapiro-Wilk test, and homoscedasticity was assessed using Levene’s test through the “Descriptives” procedure. Repeated measures ANOVA was performed to evaluate total water consumption at the drinker and feeder in relation to the age of the batch. For observations of water consumption at the drinker (L/pig/day), water consumption at the feeder (L/pig/day), water-to-feed ratio (L/kg), water-to-weight-gain ratio (L/kg), the volume of waste produced (L/pig/day), the total solids content in the waste (%), and the number of medical interventions, ANOVA was conducted. Additionally, for evaluations of daily feed intake, ADG, FCR, and CV among the pens in the experiment, covariance analysis was performed using housing weight and housing CV as covariates. A significance level of 5% was adopted, and when the ANOVA and ANCOVA tests were significant, Tukey’s test was used for pairwise mean comparisons at a 5% significance level. Mortality data did not exhibit normal or homogeneous distribution and were therefore analyzed using the Kruskal-Wallis test for non-parametric data, with a 5% significance level.

## RESULTS

### Total Water Consumption

**Figure 1.**
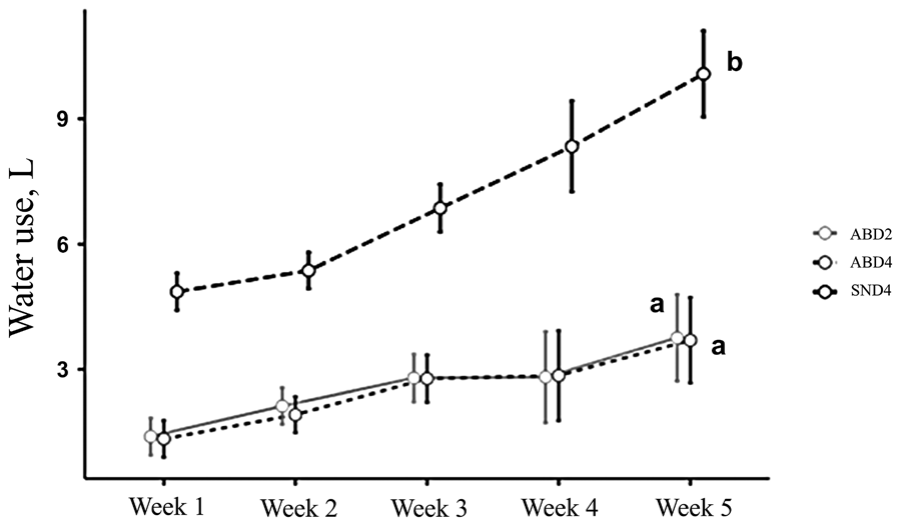
Daily water consumption at the drinker per animal in relation to the housing week. Means followed by different letters are significantly different (*P* < 0.05).

There was a difference in total water consumption at the drinkers between treatments (*P* < 0.05), but no difference in total consumption at the feeders. The swinging nipple drinker model demonstrated higher total water consumption compared to the tested bowl drinker model. During the 5 wk of the batch, the amount of water consumed in liters was higher (Figure 1). The distance between the curves in the graph in Figure 3 is greater in weeks 4 and 5, at the end of the batch, when the animals had greater age and weight. For example, in week 5, the total water consumption by animals in the SND4 treatment was, on average, 2.72 times greater than in ABD2 and ABD4. The water consumption values at feeders and drinkers are summarized in Table 1. The daily water consumption per animal in the SND4 treatment was higher than in the ABD2 and ABD4 treatments (Figure 1) for each week of age (*P* < 0.0001). In the last week of the batch, the average daily water consumption in the SND4 group reached 10.5 liters, while in the ABD2 and ABD4 groups, it was 3.92 and 3.79 liters, respectively, representing 2.77 times more than the ABD4 treatment and 2.68 times more than ABD2. Over the entire period, the average water consumption at the ABD drinkers was approximately 64% lower than at the SN drinkers (*P* < 0.0001). Similarly, the amount of water required to consume 1 kg of feed and to gain 1 kg of live weight was higher in SND4 compared to ABD2 and ABD4 (*P *< 0.0001).

**Table 1.** Total water consumption at the drinker and feeder per week of housing and the water consumption-to-feed intake and water consumption-to-weight gain ratios

	ABD2	ABD4	SND4	*P*
Week 1				
Water use in the drinker, L/pig/day	1,39 ± 0,281a	1,34 ± 0,181a	4,86 ± 0,953b	<0,0001
Water use in the feeder, L/pig/day	0,188 ± 0,072	0,184 ± 0,085	0,193 ± 0,125	0,985
Week 2				
Water use in the drinker, L/pig/day	2,25 ± 0,696a	1,96 ± 0,231a	5,62 ± 0,684b	<0,0001
Water use in the feeder, L/pig/day	0,564 ± 0,098	0,486 ± 0,154	0,515 ± 0,276	0,503
Week 3				
Water use in the drinker, L/pig/day	2,89 ± 0,569a	2,81 ± 0,421a	6,86 ± 1,14b	<0,0001
Water use in the feeder, L/pig/day	1,03 ± 0,201	0,901 ± 0,123	0,918 ± 0,352	0,354
Week 4				
Water use in the drinker, L/pig/day	2,99 ± 0,604a	2,86 ± 0,38a	8,52 ± 2,48b	<0,0001
Water use in the feeder, L/pig/day	1,29 ± 0,232	0,983 ± 0,285	1,07 ± 0,22	0,082
Week 5				
Water use in the drinker, L/pig/day	3,92 ± 0,855a	3,79 ± 0,633a	10,5 ± 2,15b	<0,0001
Water use in the feeder, L/pig/day	1,52 ± 0,225	1,34 ± 0,403	1,52 ± 0,424	0,565
Entire period				
Water use in the drinker, L/pig/day	2,69 ± 0,548a	2,55 ± 0,342a	7,27 ± 0,95b	<0,0001
Water use in the feeder, L/pig/day	0,917 ± 0,119	0,779 ± 0,173	0,843 ± 0,258	0,223
Water/feed ratio, L/kg	6,4 ± 0,63a	6,02 ± 0,71a	13,85 ± 1,86b	<0,0001
Water/weight gain ratio, L/kg	8,46 ± 1,09a	7,75 ± 0,8a	18,22 ± 2,32b	<0,0001

Different letters in the same line indicate difference between treatments (*P* < 0.05). *P = *Probability.

**Figure 2.**
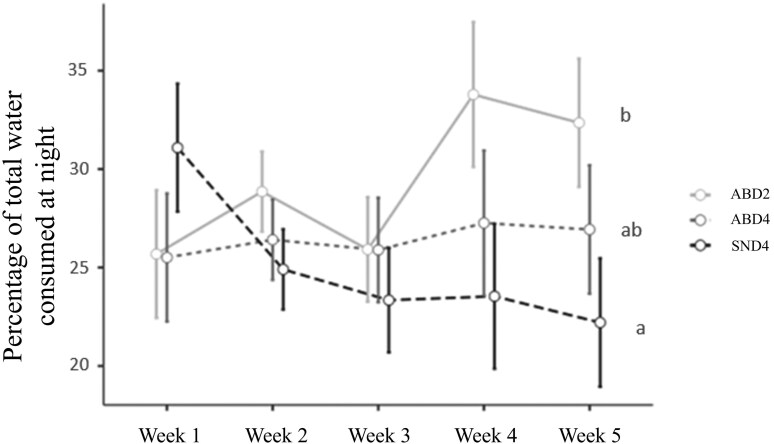
Percentage of total water consumption during the nighttime period (7:00 PM–7:00 AM) relative to the 24-hour daily period during the housing weeks. Means followed by different letters are significantly different (*P* < 0.05).

There was also a difference in the percentage of total water consumption at the drinking and feeding stations during the nighttime period (7:00 PM to 7:00 AM) compared to the full 24-hour daily period (Figure 2). This was related to an increase in this percentage during weeks 4 and 5 of housing. This percentage reached 34.2% in ABD2 animals and 25.1% in SND4 in week 4. In SND4, the rate decreased over the housing weeks. On the other hand, in ABD4, the percentage remained similar throughout the weeks.

### Manure

**Figure 3.**
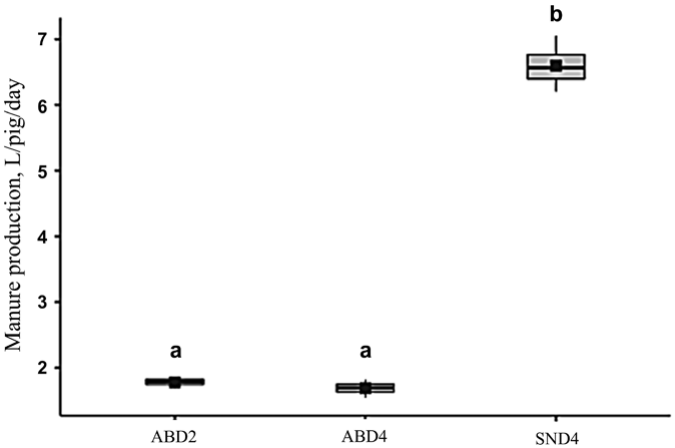
Volume of liquid manure produced per pig/day by treatments. Means followed by different letters are significantly different (*P* < 0.05).

**Figure 4.**
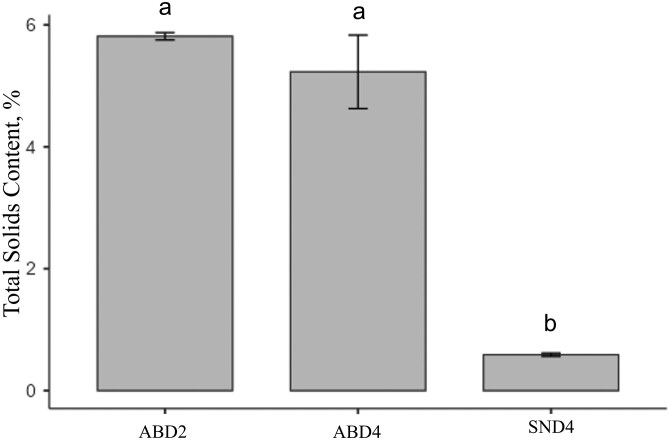
Total solids content (%) in pig liquid waste by treatment. Means followed by different letters are significantly different (*P* < 0.05).

The volume of liquid manure produced per pig per day was higher when the animals were subjected to the SN drinker model (*P* < 0.001). The volume of waste produced by the animals in the treatments with ABD drinkers was approximately 74% lower (Figure 3), being 1.78 L (± 0.07) in ABD2, 1.69 L (± 0.12) in ABD4, and 6.59 L (± 0.36) in SND4. Furthermore, the total solids content was 90% lower in the waste from the SND4 treatment (0.589% ± 0.091) compared to ABD2 and ABD4 (figure 4), which were 5.81% (± 0.2) and 5.23% (± 2.09), respectively (*P* < 0.001).

### Growth Performance, Mortality and Medical Intervention

In Table 2, it can be observed that ABD4 showed no difference in the average daily gain (ADG) of the animals compared to SND4 (*P* > 0.05). However, when the stocking density per drinker was higher (ABD2), the ADG from the midpoint to the end of the period and for the batch as a whole was lower (*P* < 0.05). There was no difference in feed conversion ratio or daily feed intake among the animals across the different treatments during the housing periods (*P *> 0.05). The coefficient of variation (CV) for final weight was lower in the ABD4 animals compared to SND4 (*P* < 0.05) and similar to ABD2 (*P* > 0.05). Final weight was higher in the SND4 animals compared to ABD2 (*P* < 0.05) and similar to ABD4 (*P* > 0.05). Although the averages for ABD2 and ABD4 were numerically similar, in the covariance analysis performed, the initial weight made the difference in final weight for ABD2 animals significant when compared to SND4 due to the numerical difference in initial weight among the animals in the treatments.

**Table 2.** Growth performance of the animals in the treatments as a function of the drinkers and stocking densities used

	ABD2	ABD4	SND4	*P*
Initial weight, kg	8,32 ± 0,332	8,17 ± 0,545	8,12 ± 0,399	0,544
Average daily gain, kg				
0 to 16 d	0,302 ± 0,043	0,304 ± 0,021	0,300 ± 0,024	0,892
16 to 35 d	0,541 ± 0,031b	0,543 ± 0,038ab	0,572 ± 0,045a	0,023
0 to 35 d	0,432 ± 0,016b	0,434 ± 0,026ab	0,454 ± 0,032a	0,0296
Feed conversion ratio				
0 to 16 dias	1,16 ± 0,092	1,10 ± 0,102	1,15 ± 0,093	0,423
16 to 35 dias	1,40 ± 0,112	1,38 ± 0,052	1,38 ± 0,033	0,913
0 to 35 dias	1,32 ± 0,068	1,29 ± 0,036	1,32 ± 0,037	0,291
Daily feed intake, kg				
0 to 16 dias	0,348 ± 0,038	0,331 ± 0,031	0,345 ± 0,033	0,564
16 to 35 dias	0,713 ± 0,043	0,707 ± 0,035	0,757 ± 0,058	0,156
0 to 35 dias	0,567 ± 0,04	0,555 ± 0,03	0,591 ± 0,05	0,245
CV peso final, %	17,8 ± 0,04ab	15,5 ± 0,02a	18,5 ± 0,02b	0,027
Peso final, kg	23,4 ± 0,778b	23,4 ± 1,37ab	24 ± 1,37a	0,029

Different letters in the same line indicate difference between treatments (*P* < 0.05). *P = *Probability.

The animals were primarily medicated for respiratory, enteric, and locomotor diseases. There was no difference (*P* > 0.05) in mortality rates, which were 0.27% (± 0.77), 0.543% (± 0.77), and 1.36% (± 1.62) for the ABD2, ABD4, and SND4 animals, respectively. The number of medical interventions per pen was 31 (± 12) for ABD2, 27.3 (± 3.58) for ABD4, and 35 (± 12.1) for SND4. There was no difference in the number of medical interventions per pen (*P* > 0.05).

## DISCUSSION

### Total Water Use

The pendular nipple drinker model demonstrated higher total water usage compared to the tested bowl-type drinker model. As shown in Figure 1, water consumption in liters was higher across all 5 wk of the batch. The gap between the curves in the graph increased during weeks 4 and 5, coinciding with the end of the batch and the animals’ larger size and weight. For instance, in week 5, the total water consumption by the SND4 animals was, on average, 2.72 times higher than that of ABD2 and ABD4. Phillips and Phillips (1999) observed lower waste percentages with another bowl-type drinker model compared to fixed nipple drinkers during the first week of the nursery phase (19% and 57% for the bowl and nipple models, respectively). Similarly, Torrey et al. (2008) compared bowl drinkers with trigger pins and float mechanisms, which also demonstrated lower waste percentages than fixed nipple models (19%, 39%, and 57%, respectively). Moreover, Meizhi et al. (2017) also reported lower waste with the bowl model; however, when comparing fixed nipple to pendular nipple models, they found the pendular model exhibited a lower waste percentage (15%, 25%, and 18%, respectively). This lower waste in pendular nipple drinkers can be correlated with reduced total water consumption and, consequently, a lower volume of waste produced per pig per day, as observed by Brumm et al. (2000) during the growing phase when they compared the 2 nipple drinker models. In a more recent study, Vande Pol et al. (2022) compared total water usage between a fixed nipple drinker and a bowl drinker mounted on a wall with a nipple trigger. They observed approximately 18% lower water usage with the bowl model. Thus, water waste is directly related to the drinker model. The present study supports the findings of Brumm et al. (2000), which showed that drinkers with a reservoir for water consumption lead to lower total water usage and waste. However, factors such as stocking density, drinker design, and location within the pen influence animal behavior (Magowan et al., 2007), which in turn affects the magnitude of the differences in total water consumption and waste between the models tested.

Another factor that directly impacts water waste is the flow rate of nipple drinkers. Higher flow rates increase the percentage of water waste (Nienaber and Hahn, 1984). In this study, the flow rate used (1,216 ± 0.257 mL/min) for the nipples exceeded the recommended rate for piglets in the nursery phase (Patience, 2012). However, this is a common practice aimed at increasing water intake and, consequently, feed consumption (Barber et al., 1989). Greater alignment between technical recommendations from drinker suppliers and further research is necessary to enhance understanding of the relationship between flow rate, consumption, and water waste (Magowan et al., 2007; Vande Pol et al., 2022). The use of dry/wet feeders improves feed intake and, consequently, performance, particularly in animals during the growing and finishing phases (Bergstrom et al., 2012). The use of water in feeders is widely employed in nursery facilities to achieve similar gains observed in later phases. Despite the higher water consumption by animals in the SND4 treatment, water use in the dry/wet feeder showed no compensatory effect on feed intake, indicating that the higher water consumption with the nipple drinker is related to waste rather than effective consumption.

The bowl drinker model tested in this study did not require cleaning during the experimental period. Daily checks were performed to assess the need for cleaning, as a clean drinker in the days immediately after weaning increases total water consumption (Phillips and Phillips, 1999). Additionally, excreta and feed accumulation can act as contaminants for the animals. The bowl’s placement near the center of the pen and its low, constant water level contributed to maintaining its cleanliness.

In summary, the higher total water usage observed with the pendular nipple drinker in this study does not necessarily indicate greater effective water consumption by the animals, but rather a higher level of water waste during drinking. While the drinker model did not influence zootechnical performance, it significantly affected the efficiency of water delivery. The pendular nipple system, especially when combined with a high flow rate, allowed greater spillage, particularly as the animals grew larger toward the end of the nursery phase. Conversely, the bowl drinker demonstrated reduced waste and maintained consistent water availability without compromising hygiene. Therefore, the choice of drinker should consider not only water availability but also waste minimization, which is critical for sustainable management practices and environmental impact reduction in swine production systems.

### Volume and Concentration of the Manure

Since the volume of waste produced by animals in ABD2 and ABD4 was approximately 74% lower than in SND4, and the total solids content was 90% lower in SND4, it can be concluded that waste is higher in nipple drinkers, as observed in previous studies. The daily liquid waste volume per pig (liters) observed in this experiment for ABD2 and ABD4 was close to the values reported in Normative Instruction No. 11 of the Santa Catarina Environmental Institute (2021) (1.78, 1.69, and 1.60, respectively). However, in SND4, this value was 6.59 L/pig/day, indicating a significantly higher volume, resulting in greater environmental impact, higher costs, and lower nutrient utilization from this waste. Vande Pol et al. (2022) demonstrated that the water-to-feed ratio increased from 4.22 to 5.23 L/kg when cup drinkers were replaced by nipple drinkers, without any impact on animal performance indicating water wastage. Brumm et al. (2000), studying animals in the growing phase, found a liquid waste volume of 3.96 L/pig/day using pendular nipple drinkers, which was lower than the value found in this study for the same drinker model. This difference is directly related to the lower flow rate and the animal age range used in each experiment.

Studies in grow-finish phases confirm that this excess water increases pit volume by 13 to 40% (Chastain et al., 1998; Brumm et al., 2000). In practice, this requires larger storage tanks, more frequent removal cycles, higher energy consumption for pumping, and greater CO₂ emissions associated with transporting a lower-density nutrient material. It also complicates processes such as solid-liquid separation or concentration via reverse osmosis. In high-standard European environmental systems, a 1 L increase per pig per day in manure volume can raise annual storage and application costs by 4% to 6%, especially in regions with regulated nitrogen application limits per hectare (Velthof et al., 2019).

Using an example of pig liquid manure with 3% dry matter, it can be stated that its composition per cubic meter consists of 2.8 kg of nitrogen, 2.4 kg of phosphorus, 1.5 kg of potassium, and smaller amounts of calcium, magnesium, and other nutrients (Silva et al., 2016). Therefore, the higher the total solids content, the greater the nutrient concentrations. Conversely, the greater the dilution, or moisture content, the lower the nutrient concentration. Performing a simple proportional calculation on a sample of waste from the ABD2 and ABD4 treatments would have, on average, 5.15 kg of nitrogen, 4.42 kg of phosphorus, and 2.76 kg of potassium per cubic meter. In contrast, a sample from SND4 would have 0.549 kg of nitrogen, 0.47 kg of phosphorus, and 0.29 kg of potassium per cubic meter.

In fertigation, nutrient concentration differences directly affect agronomic efficiency. With drinkers that minimize water wastage, such as the ABD model tested, the average estimated nitrogen concentration of 5.15 kg N m⁻³ enables the application of 100 kg N ha⁻¹ with only 19 m³ ha⁻¹ compatible with irrigation depths of approximately 2 mm. In contrast, the diluted slurry produced by nipple drinkers (0.55 kg N m⁻³) would require ~180 m³ ha⁻¹ to deliver the same nitrogen dose. This volume often exceeds the water-holding capacity of many soils, increasing the risk of phosphorus and potassium leaching and demanding more operational time (Rosa et al., 2017). Therefore, the use of drinkers that reduce water wastage is advantageous, as they result in manure with higher nutrient concentrations. This enables the application of lower volumes per hectare to achieve the same nitrogen dose, optimizing management, reducing transportation and application costs, and improving the efficiency and sustainability of fertigation.

The ABD model tested in this experiment provided greater sustainability and better waste quality, which can be easily converted into structural, environmental, and financial benefits for a farm.

Evaluating water waste and waste production in a commercial farm is not part of routine practices because it requires specific structural conditions and labor. However, the indirect method using the total solids content technique proved to be feasible and is a useful tool for commercial farms and research projects to assess water waste.

### Growth Performance

No significant differences in average daily gain (ADG) were observed among treatments during the first 16 d of the batch. However, from day 16 to 35 and when evaluating the overall period, animals in SND4 showed better ADG than those in ABD2. In the ABD2 pens, the density was 23 animals per drinker reservoir, effectively doubling the number of animals per water source. In ABD4, although a different model from SND4 was used, the density was the same, resulting in no significant difference in ADG (*P* < 0.05). This difference in ADG contributed to the higher final weight of animals in SND4 compared to those in ABD2. Jackson et al. (2021) observed that a lower density of animals per drinker resulted in a higher number of visits and more time spent at the drinker, indicating that reduced competition facilitates access to and increases water consumption. Additionally, as shown in Figure 2, the percentage of total nighttime water use increased during the final 2 wk of the batch in ABD2, a trend not similarly observed in ABD4 and SND4. Daytime water use (from 7:00 AM to 7:00 PM) still accounted for the majority of total water usage, as in week 4, for example, 34.2% of water use in ABD2 occurred at night, while the remaining 65.8% occurred during the day. The increased proportion of nighttime water use is linked to greater competition, as heavier animals with higher daily water requirements experience restricted access. Thus, doubling the number of animals per water reservoir is associated with the lower ADG observed in ABD2 animals.

No differences were found in feed conversion rates or daily feed intake across treatments. In studies with the same animal densities between drinker models, no differences were found in ADG, feed conversion, or daily feed intake either (Phillips and Phillips, 1999; Brumm et al., 2000; Magowan et al., 2007; Torrey et al., 2008; Vande Pol et al., 2022).

The water-to-feed intake ratio and the water-to-weight gain ratio (Table 2) were higher in SND4 animals than in ABD2 and ABD4 animals (*P* < 0.0001). In both evaluations, the bowl drinker demonstrated approximately 55% lower ratios. Brumm et al. (2000) and Vande Pol et al. (2022) also found lower water usage per kilogram of feed consumed in bowl drinker models compared to fixed nipple drinkers, with no differences in ADG.

The coefficient of variation (CV) of final delivery weight (%) was lower in ABD4 animals compared to SND4 animals. Thus, when comparing the ABD model used in this study to the SN model with the same number of animals per drinker, the weight variation within pens was lower, indicating more uniform delivery weights. Easy water access and rapid consumption, especially early in the batch, provided by the ABD model, are related to the lower CV in final weight. The bowl drinker acts as a reservoir that pigs discover sooner after weaning (Phillips and Fraser, 1991). Moreover, this drinker does not require activation by the animal and constantly provides water, with the visible water layer mimicking natural drinking behavior (Meunier-Salaün et al., 2017). Consequently, competition for water consumption is reduced, as newly weaned piglets quickly learn how to drink, and ideal drinker density ensures easy and quick access, minimizing competition. Improved weight uniformity within pens, as observed with the use of the bowl drinker (ABD model), has important implications beyond the nursery phase. Greater uniformity facilitates more efficient grouping strategies for the finishing phase, as animals with similar weights can be housed together, optimizing space use, feeding strategies, and health management (Maselyne et al., 2015). This consistency reduces the number of sorting events needed throughout the production cycle and simplifies the logistics of transport and slaughter scheduling. Uniform batches improve slaughterhouse efficiency by reducing carcass weight variability, which is critical for meeting commercial specifications and maximizing revenue per animal. Furthermore, precise slaughter planning leads to better alignment between farm production and processing capacity, enhancing the overall productivity and profitability of the pork supply chain. Thus, management interventions that promote early water intake and improve uniformity such as the adoption of bowl drinkers with adequate drinker-to-animal ratios should be considered strategic tools for optimizing production systems.

Due to the constant water layer available to animals, the bowl drinker model used in ABD2 and ABD4 presented a particular behavior on hotter days. While in pens with pendular nipple drinkers, animals activated the nipple to wet themselves and improve their environment, in ABD2 and ABD4 pens, animals lay near the drinker, often with their snouts in the water. As a result, most of the animals in the pen lacked access to water, especially in ABD2, which had only 2 bowls. This behavior further contributed to the ADG differences.

There were no differences in the number of medical interventions or mortality rates (%) among treatments (*P* > 0.05). On average, Brazil uses 358 mg of antimicrobial active ingredients per kilogram of pork produced (Dutra, 2017), with most medications administered via water and consequently incorporated into waste. This practice not only leads to the generation of multi-resistant strains that can spread in the environment and food production chain (Pissetti, unpublished results), directly affecting public health but also inhibits plant growth due to impacts on soil microbiota, the ultimate destination for waste (Migliore et al., 2003). Reducing the indiscriminate use of antimicrobials, combined with minimizing medicated water waste, drastically decreases the environmental impact of this contamination and general medication costs, as higher water waste necessitates compensatory dosing.

Therefore, avoiding the waste of medicated water into the environment is a crucial step toward sustainable pig farming. Studies have shown that up to 50% of antimicrobials administered via drinking water may not be consumed by animals and are instead excreted or spilled, ending up in manure and wastewater (Zhou et al., 2013; Berendsen et al., 2015). This contaminated effluent, when applied as fertilizer, introduces active pharmaceutical residues into the soil and aquatic systems, where they persist and accumulate, promoting antimicrobial resistance and disrupting native microbial communities essential for nutrient cycling and plant health (Topp et al., 2013). Moreover, the environmental half-life of some compounds, such as tetracyclines, can exceed 100 d in soil (Hamscher et al., 2002), emphasizing the long-term risk of improper disposal. Thus, improving precision in drug administration and optimizing water delivery systems are essential strategies to safeguard ecosystems, reduce resistance proliferation, and enhance the overall efficiency of swine production systems.

Finally, the choice of drinker model in commercial farms should not only consider its potential to enhance animal performance but also evaluate water waste, waste production, and medium- to long-term cost-effectiveness.

## Conclusion

The bowl drinker model with a maintained water layer used in this study resulted in lower total water usage and reduced manure volume, with a higher percentage of total solids. Additionally, it contributed to less variation in the final body weight of the animals without compromising zootechnical performance. This reduction in weight variability at loading potentially enhances the efficiency of the swine production chain by optimizing batch uniformity, improving slaughter scheduling, and reducing logistical and processing inefficiencies.
